# Whole-genome and pan-genome analyses reveal genomic differences among nontypeable *Haemophilus influenzae* isolates from bronchiectasis, community-acquired pneumonia, and chronic obstructive pulmonary disease

**DOI:** 10.3389/fcimb.2026.1827485

**Published:** 2026-08-07

**Authors:** Ting Hou, Qing-Hua Gao, Qiao-Li Zhang, Si-Ji Zhou, Lei Zhang, Qing-Lin He, Li-Ping Cheng, Na Zhang, Tong Guan, Yu-Yun Yang, Xiao-Ran Li, Jian He

**Affiliations:** Faculty of Life Science and Technology & The Affiliated Anning First People’s Hospital, Kunming University of Science and Technology, Kunming, China

**Keywords:** accessory genome, bronchiectasis, chronic obstructive pulmonary disease, comparative genomics, nontypeable *Haemophilus influenzae*, pan-genome-wide association analysis

## Abstract

**Background:**

Nontypeable *Haemophilus influenzae* (NTHi) is a major bacterial pathogen in both acute and chronic respiratory diseases, yet the genomic basis underlying its association with different clinical phenotypes remains incompletely understood.

**Methods:**

We performed whole-genome sequencing and comparative genomic analysis of 27 NTHi isolates, including strains derived from patients with bronchiectasis (n = 10), community-acquired pneumonia (CAP; n = 6), and chronic obstructive pulmonary disease (COPD; n = 11). Among these, three isolates (one from each disease group) were newly sequenced using a hybrid Oxford Nanopore–Illumina approach, while the remaining 24 genomes were retrieved from public databases. Pan-genome analysis, multilocus sequence typing (MLST), core genome phylogenetic analysis, accessory genome based discriminant analysis, and pan-genome-wide association analysis (pan-GWAS) were performed to characterize genomic diversity and variation in gene content across isolates.

**Results:**

MLST and core genome phylogenetic analyses revealed substantial genetic diversity, with isolates from bronchiectasis, CAP, and COPD distributed across multiple lineages without clear disease-specific clustering. Accessory genome–based analyses indicated heterogeneous differences in gene content among isolates from different clinical backgrounds, although overlap between groups remained evident. Pan-genome-wide association analysis did not identify any accessory genes that remained statistically significant after Benjamini–Hochberg false discovery rate (FDR) correction. Under a relaxed exploratory threshold (empirical P-value < 0.35 and odds ratio > 1), a subset of accessory genes showing differential distribution patterns among disease groups was identified and reported as exploratory candidates. Comparatively greater accessory genome divergence was observed between bronchiectasis and COPD isolates. Functional annotation indicated that these candidate genes spanned multiple categories, including recombination, membrane-associated processes, transport, and nutrient utilization.

**Conclusions:**

Clinical heterogeneity among NTHi isolates was not reflected in core genome phylogeny. Differences in accessory gene content were observed across isolates from different clinical sources; however, these patterns were not supported by statistically robust associations after multiple testing correction. The identified candidate genes should therefore be regarded as exploratory, and the findings interpreted as descriptive of genomic diversity rather than evidence of disease-associated functional differentiation.

## Introduction

Nontypeable *Haemophilus influenzae* (NTHi) is a common commensal of the human upper respiratory tract and an important opportunistic pathogen associated with a broad spectrum of airway diseases. Although colonization is often asymptomatic, disruption of host immune homeostasis can facilitate NTHi invasion into the lower respiratory tract, contributing to clinical conditions ranging from acute community-acquired pneumonia (CAP) to chronic airway diseases such as bronchiectasis and chronic obstructive pulmonary disease (COPD) (Agrawal and Murphy, 2011; Hare et al., 2012; Shahid et al., 2021; Slack, 2015). Bronchiectasis, CAP, and COPD are clinically and biologically heterogeneous disorders, characterized by diverse inflammatory endotypes, microbial communities, and patterns of disease progression, in which chronic bacterial infection plays a central pathogenic role (De Angelis et al., 2025; Garmendia et al., 2014; Murphy et al., 2004).

Data from the European Bronchiectasis Registry (EMBARC) demonstrate pronounced regional variation in airway microbiology across Europe, with *Haemophilus influenzae* being more frequently isolated in northern and western Europe, including the United Kingdom, whereas *Pseudomonas aeruginosa* predominates in southern European countries (Spinou et al., 2024). Sara E. Oliver et al. (2023) reported that NTHi has become the leading cause of invasive *H. influenzae* disease, accounting for more than 78% of all reported invasive cases, with an average annual increase of 7.4% between 2007 and 2014. Consistent with these global and regional trends, our group conducted a retrospective etiological surveillance of patients with bronchiectasis in Yunnan Province, China. Among pathogens detected during acute exacerbations, NTHi accounted for 26.83% of isolates, significantly exceeding the prevalence of *P. aeruginosa* (14.88%) (Huang et al., 2025). Likewise, NTHi is a frequent bacterial cause of COPD exacerbations and an established etiologic agent of lower respiratory tract infection (Shahid et al., 2021). However, despite overlapping microbiological features, bronchiectasis and COPD differ substantially in airway architecture, inflammatory milieu, and chronicity of infection, suggesting that bacterial adaptation strategies may not be uniform across these disease contexts (Athanazio, 2012; Mac et al., 2024).

The remarkable ability of NTHi to persist across diverse airway environments is largely attributable to its extensive genetic diversity (Garmendia et al., 2012). While the core genome is relatively conserved, NTHi possesses a highly dynamic accessory genome shaped by frequent recombination, horizontal gene transfer, and within-host evolutionary pressures (Harrison et al., 2020; Messer et al., 2017; Wilson et al., 2016). These processes generate substantial phenotypic variability, influencing nutrient acquisition, biofilm formation, immune evasion, and long-term colonization (Duell et al., 2016). Population genomic studies have shown that NTHi strains form multiple phylogenetic lineages, yet isolates from different clinical backgrounds are often intermingled within core genome–based trees (Kc et al., 2020; Pettigrew et al., 2018). Consequently, traditional genotyping approaches, including MLST and core-genome SNP phylogenies, have failed to reliably link specific genotypes to disease phenotypes (De Chiara et al., 2014; LaCross et al., 2013).

Increasing evidence suggests that variation within the accessory genome plays a critical role in niche adaptation and disease behavior (Abdelbary et al., 2024; Dahiya et al., 2024; Torres-Cruz and van der Woude, 2003). In this context, whole-genome analyses have begun to reveal clinically relevant gene content differences among NTHi isolates. Notably, a previous study focusing on COPD demonstrated that NTHi isolates from COPD patients harbor distinct accessory gene repertoires compared with isolates from other clinical phenotypes (Kc et al., 2020). However, despite the recognized clinical importance of NTHi in bronchiectasis, comparable genome-wide analyses specifically targeting bronchiectasis-associated NTHi isolates remain lacking. Thus, the genomic features that may underlie NTHi adaptation to the chronically inflamed and structurally damaged airways of bronchiectasis patients are still poorly understood.

In this study, we performed comprehensive genomic characterization of 27 NTHi isolates derived from patients with bronchiectasis, CAP and COPD. By integrating whole-genome sequencing, pan-genome analysis, core genome phylogenetic analysis, accessory genome population structure analysis, and pan-GWAS, we characterized patterns of genomic variation and differences in accessory gene composition among isolates from distinct clinical sources. This study was designed as an exploratory genomic investigation aimed at describing genomic diversity across NTHi isolates from different clinical backgrounds and providing a comparative framework for future studies of NTHi population structure in respiratory disease.

## Methods

The collection and use of clinical isolates in this study were approved by the Ethics Committee of The Affiliated Anning First People’s Hospital of Kunming University of Science and Technology (Approval No. 2023-077-01). All isolates were obtained as part of routine clinical care, and all patient data were anonymized prior to analysis.

This study analyzed a total of 27 NTHi isolates, including three newly sequenced strains collected at the same institution (one isolate each from patients with bronchiectasis, CAP, and COPD) and 24 publicly available NTHi genomes retrieved from the NCBI RefSeq database (Dataset 1). Clinical metadata were used to classify all isolates into three disease groups: bronchiectasis (n = 10), CAP (n = 6), and COPD (n = 11) (**Table 1**). The whole-genome shotgun sequences of the three newly sequenced isolates have been deposited in GenBank under the accession numbers JBUBLW000000000, JBUBLX000000000, and JBUBLY000000000 (He, 2025a; He, 2025b; He, 2025c).

**Table 1:** Information and clinical grouping of *H. influenzae* strains used in this study.

Group	Strain	Assembly	Site of isolation	Clinical origin	Collection site	Serotype	Source	Reference
Bronchiectasis group (n=10)	B1	GCA_054901855.1	bronchoalveolar lavage	BE	China: Kunming, Yunnan	NTHi	This study	–
	60295_BAL_Hi1	GCA_004802095.1	bronchoalveolar lavage	BE	Australia: Northern Territory	NTHi	NCBI -	(Aziz et al., 2019)
	60316_BAL_Hi1	GCA_004801985.1	bronchoalveolar lavage	BE	Australia: Northern Territory	NTHi	NCBI -	(Aziz et al., 2019)
	65290_BAL_Hi4	GCA_004802155.1	bronchoalveolar lavage	BE	Australia: Queensland	NTHi	NCBI -	(Aziz et al., 2019)
	60294N1	GCA_000818925.1	bronchoalveolar lavage	BE	Australia: Northern Territory	NTHi	NCBI	–
	60362_NP_Hi1	GCA_004802395.1	nasopharynx	BE	Australia: Northern Territory	NTHi	NCBI	(Aziz et al., 2019)
	60295_NP_Hi1	GCA_004802075.1	nasopharynx	BE	Australia: Northern Territory	NTHi	NCBI	(Aziz et al., 2019)
	65290_NP_Hi3	GCA_004802225.1	nasopharynx	BE	Australia: Queensland	NTHi	NCBI	(Aziz et al., 2019)
	60069_NP_Hi2	GCA_004802085.1	nasopharynx	BE	Australia: Northern Territory	NTHi	NCBI	(Aziz et al., 2019)
	60294_NP_Hi1	GCA_004802405.1	nasopharynx	BE	Australia: Northern Territory	NTHi	NCBI	(Aziz et al., 2019)
CAP group (n=6)	P1	GCA_054901975.1	nasopharynx	PN	China: Kunming, Yunnan	NTHi	This study	–
	Hi642	GCA_003363355.1	blood	PN	Italy: Marche	NTHi	NCBI	(Watts and Holt, 2019)
	Hi361	GCA_000833815.1	blood	PN	Italy: Milano	NTHi	NCBI	(Giufrè et al., 2015)
	444/18	GCA_020073685.1	bronchoalveolar lavage	PN	Poland: Warszawa	NTHi	NCBI	–
	146	GCA_002917845.1	blood	PN	Brazil	NTHi	NCBI	–
	CCUG 26214	GCA_001679235.1 (Project, 2016)	nasopharynx	PN	USA: Massachusetts	NTHi	NCBI	–
COPD group (n=11)	C1	GCA_054901955.1	nasopharynx	COPD	China: Kunming, Yunnan	NTHi	This study	–
	P669-6977	GCA_003425505.1	nasopharynx	COPD	Spain: Catalunya	NTHi	NCBI	–
	P665-7858	GCA_003425645.1	nasopharynx	COPD	Spain: Catalunya	NTHi	NCBI	–
	6P32H1	GCA_002966715.1	nasopharynx	COPD	USA: Buffalo, NY	NTHi	NCBI	(Pettigrew et al., 2018)
	5P28H1	GCA_002966615.1	nasopharynx	COPD	USA: Buffalo, NY	NTHi	NCBI	(Pettigrew et al., 2018)
	P676-2514	GCA_003425605.1	nasopharynx	COPD	Spain: Catalunya	NTHi	NCBI	(Pettigrew et al., 2018)
	P641-4342	GCA_003425935.1	nasopharynx	COPD	Spain: Catalunya	NTHi	NCBI	–
	P632-8384	GCA_003415085.2	nasopharynx	COPD	Spain: Catalunya	NTHi	NCBI	–
	87P37H1	GCA_002988835.1	nasopharynx	COPD	USA: Buffalo, NY	NTHi	NCBI	–
	107P75H1	GCA_002989275.1	nasopharynx	COPD	USA: Buffalo, NY	NTHi	NCBI	–
	19P49H1	GCA_002989925.1	nasopharynx	COPD	USA: Buffalo, NY	NTHi	NCBI	–

BE, Bronchiectasis; PN, CAP, Community-acquired pneumonia; COPD, Chronic Obstructive Pulmonary Disease.

### Genome assembly, polishing, and quality assessment

To obtain high-quality complete genomes, hybrid assembly was performed. ONT long reads were first assembled using Canu v2.0, producing initial draft assemblies. Long-read polishing was conducted using Racon v1.4.13 (three iterations), followed by short-read error correction using Pilon v1.22 (three iterations) (Gautam et al., 2019).

Genome quality and completeness were evaluated using CheckM v1.2.3, and sequencing depth metrics were calculated using Illumina read mapping. All assemblies were further aligned to the complete genome of NTHi strain 86-028NP to assess genome integrity and coverage. Assembly quality metrics, including genome size, scaffold number, N50, completeness, and sequencing coverage, are summarized in **Table 2**.

**Table 2:** Assembly statistics.

Strain	Assembly	Seq type	Genome size(Mb)	Total number	N50 length(kb)	Genome coverage	GC content(%)	Completeness(%)	Sequencing platform
B1	B1.1	Complete genome	1.8	1	1813.13	1,012x	38.08	99.66	ONT; Illumina MiSeq
60295_BAL_Hi1	GCA_004802095.1	Scaffold	1.8	11	347.6	335x	38	99.66	Illumina NextSeq
60316_BAL_Hi1	GCA_004801985.1	Scaffold	1.9	19	245.5	343x	38	98.87	Illumina HiSeq
65290_BAL_Hi4	GCA_004802155.1	Scaffold	1.8	10	1000	297x	38	99.67	Illumina NextSeq
60294N1	GCA_000818925.1	Scaffold	1.8	19	262.4	100x	–	99.00	Illumina MiSeq
60362_NP_Hi1	GCA_004802395.1	Scaffold	1.9	8	387	315x	38	98.52	Illumina HiSeq
60295_NP_Hi1	GCA_004802075.1	Scaffold	1.8	15	352.3	354x	38	99.66	Illumina HiSeq
65290_NP_Hi3	GCA_004802225.1	Scaffold	1.7	7	1000	354x	37.5	99.65	Illumina HiSeq
60069_NP_Hi2	GCA_004802085.1	Scaffold	1.8	19	222.2	311x	38	98.99	Illumina HiSeq
60294_NP_Hi1	GCA_004802405.1	Scaffold	1.8	19	262.4	312x	38	99.64	Illumina HiSeq
P1	P1.1	Complete genome	2.0	1	1880.45	727x	39.57	99.77	ONT; Illumina MiSeq
Hi642	GCA_003363355.1	Scaffold	1.8	50	231.6	25x	38	99.67	Illumina HiSeq
Hi361	GCA_000833815.1	Scaffold	1.8	21	140.8	50x	38	99.55	Illumina HiSeq
444/18	GCA_020073685.1	Scaffold	1.8	36	403.9	50x	38	99.51	Illumina HiSeq
146	GCA_002917845.1	Scaffold	1.8	56	110.1	690.12x	38	99.37	Illumina NextSeq
CCUG 26214	GCA_001679235.1 (Project, 2016)	Scaffold	1.8	21	994.2	221x	38	98.07	Illumina MiSeq
C1	C1.1	Complete genome	2.0	1	1797.07	614x	38.04	99.67	ONT; Illumina MiSeq
P669-6977	GCA_003425505.1	Scaffold	2.0	1	2000	141x	38.5	99.55	PacBio RSII
P665-7858	GCA_003425645.1	Scaffold	1.9	1	1900	114x	38	98.96	PacBio RSII
6P32H1	GCA_002966715.1	Scaffold	1.9	1	1900	56.3583x	38	99.44	PacBio RS II;Illumina HiSeq 2000
5P28H1	GCA_002966615.1	Scaffold	1.9	1	1900	59.4537x	38	99.42	PacBio RS II;Illumina HiSeq 2000
P676-2514	GCA_003425605.1	Scaffold	1.9	1	1900	83x	38	99.42	PacBio RSII
P641-4342	GCA_003425935.1	Scaffold	1.8	1	1800	125x	38	99.55	PacBio RSII
P632-8384	GCA_003415085.2	Scaffold	1.8	37	158.8	197.443x	38	99.65	Illumina NextSeq
87P37H1	GCA_002988835.1	Scaffold	1.8	67	103.6	53.5951x	38	99.58	Illumina HiSeq 2000
107P75H1	GCA_002989275.1	Scaffold	1.8	65	105.6	127.768x	38	99.48	Illumina HiSeq 2000
19P49H1	GCA_002989925.1	Scaffold	1.9	79	87.3	47.7204x	38	99.53	Illumina HiSeq 2000

All genomes, including both newly sequenced complete genomes and publicly available draft genomes, were processed using a consistent annotation and pan-genome analysis pipeline. While this approach reduces methodological variability, differences in assembly quality—such as fragmentation, completeness, contiguity, and sequencing depth—may still influence gene prediction and accessory gene detection.

Genomes with low completeness or extreme fragmentation were to be excluded; however, all assemblies included in this study met acceptable quality criteria based on the above metrics and were therefore retained for downstream analyses. Importantly, gene absence inferred from draft genomes should be interpreted with caution, as fragmented assemblies may lead to underestimation of accessory gene content. Accordingly, potential biases in gene presence–absence inference related to assembly quality were considered in downstream analyses and interpretation.

### Genome annotation

Structural and functional annotation of assembled genomes was performed using Prokka v1.14.6 with default parameters. Coding sequences (CDSs) were aligned against UniProt and Pfam databases using an E-value cutoff of 1e-6 to identify conserved domains (Lowe and Eddy, 1997; Seemann, 2014). Genes without significant database matches were annotated as hypothetical proteins.

### Pan-genome construction

A pan-genome was constructed using Panaroo (Tonkin-Hill et al., 2020) with Prokka-derived GFF3 annotation files as input. Panaroo was run in default (strict) mode to improve gene clustering accuracy, particularly for draft genome assemblies. Gene clustering and gene presence–absence inference were performed using default parameters implemented in Panaroo.

Genes were classified based on their frequency across isolates, with core genes defined as those present in ≥ 95% of genomes, shell genes present in 15 – 95% of genomes, and cloud/unique genes present in < 15% of genomes. The resulting gene presence–absence matrix (gene_presence_absence.csv) was used for downstream analyses (Dataset 2).

### Multilocus sequence typing

MLST was performed using allelic profiles of seven housekeeping genes (*adk*, *atp*G, *frd*B, *fuc*K, *mdh*, *pgi*, *rec*A) following the PubMLST *H. influenzae* scheme (Jolley et al., 2018). STs were assigned using the PubMLST database. A MST was constructed using the goeBURST algorithm and visualized with PHYLOViZ v2.0 (Francisco et al., 2009, 2012).

### Phylogenetic reconstruction

Core genome alignment was generated prior to phylogenetic analysis. A maximum-likelihood (ML) phylogenetic tree was reconstructed using IQ-TREE v2.2.0 based on the aligned core genome sequences (Minh et al., 2020). The optimal nucleotide substitution model was automatically selected using ModelFinder (Kalyaanamoorthy et al., 2017), and branch support was evaluated using 1,000 ultrafast bootstrap replicates implemented in UFBoot2 (Hoang et al., 2018). The resulting phylogeny was exported in Newick format and visualized using the Interactive Tree of Life (iTOL) v6 platform (Letunic and Bork, 2021).

### Accessory-genome DAPC analysis

Accessory genome–based discriminant analysis of principal components (DAPC) was performed using the adegenet package in R (Jombart, 2008). Presence–absence matrices of accessory genes were derived from the Panaroo-generated gene_presence_absence.csv file for all isolates to ensure consistency in gene clustering and annotation. Principal component analysis (PCA) was used as a prior dimensionality reduction step. The number of genetic clusters was explored using k-means clustering implemented in find.clusters, with model selection guided by the Bayesian Information Criterion (BIC) (Li et al., 2002), which supported four clusters. To evaluate clustering robustness and reduce overfitting, cross-validation was performed using the xvalDapc function with 100 replicates and a training proportion of 90%. The optimal number of retained principal components (PCs) was determined based on the highest mean assignment success. Based on this procedure, 10 PCs were retained, yielding a mean assignment success of approximately 85%. Retaining additional PCs did not substantially improve assignment success but increased variability among replicates, suggesting potential overfitting, whereas fewer PCs resulted in reduced assignment accuracy. DAPC was then performed using the retained PCs, and the first two discriminant functions were used for visualization. These analyses were exploratory and intended for descriptive comparison of accessory genome variation (Jombart et al., 2010).

### Pan-genome wide association study

A pan-genome wide association study (pan-GWAS) was performed using Scoary v1.6.16 (Brynildsrud et al., 2016). The gene presence–absence matrix generated by Panaroo v1.5.0 (Tonkin-Hill et al., 2020) under default parameters was used as the input genotype matrix. Clinical phenotypes (bronchiectasis, CAP, and COPD) were encoded as binary traits in a trait.csv file for association testing.

For each accessory gene, Fisher’s exact test was used to assess phenotype–genotype associations, and odds ratios were calculated as measures of effect size. P-values were adjusted for multiple testing using the Benjamini–Hochberg FDR method, and genes with FDR-adjusted *P* < 0.05 were considered statistically significant.

Given the relatively small and heterogeneous isolate collection, which may limit statistical power, no genes remained statistically significant after multiple-testing correction. Therefore, additional exploratory analyses were performed using a more relaxed threshold (empirical P-value < 0.35 and odds ratio > 1) to identify candidate genes for descriptive and hypothesis-generating purposes only. These exploratory results were not considered statistically significant and were not interpreted as evidence of disease-associated genes.

### Functional annotation of exploratory candidate genes

A multi-database annotation strategy was used to interpret the biological functions of exploratory candidate genes showing differential distribution patterns across respiratory phenotypes. Prokka-predicted CDSs were translated into amino acid sequences. BLASTP searches were performed against the UniProt database (E < 1e-3, identity ≥ 70%).Functional classification was performed using the Clusters of Orthologous Groups (COG) database (Galperin et al., 2015; Reiner et al., 2018), KEGG pathway annotations (Kanehisa et al., 2004), and the PANTHER classification system (v14.0) (Mi et al., 2019). Protein domains and Gene Ontology (GO) terms were identified using InterProScan v5.52-86.0. Candidate genes were classified according to molecular function, biological process, and cellular component (Ashburner et al., 2000).

## Results

### Genome sequencing and assembly overview

In this study, three NTHi isolates were sequenced using Oxford Nanopore technology, generating an average of approximately 1.48 Gb of raw data per isolate. Following quality control, an average of 1.42 Gb of high-quality clean data was retained for each isolate. All three genomes were successfully assembled into complete circular chromosomes. The assembled genomes exhibited a mean length of 1.97 Mb and an average GC content of 38.56%, with adequate sequencing depth. Additional assembly metrics, including sequencing strategy, genome size, scaffold number, N50, completeness, genome coverage, and sequencing platform, are summarized in **Table 2**. Genome coverage varied substantially among isolates, ranging from approximately 25× to over 1000×, primarily reflecting differences in sequencing platforms, library preparation strategies, and data sources. Despite this variability, all genomes included in this study met predefined quality criteria, with assembly statistics falling within acceptable ranges for comparative genomic analysis. In addition, 24 publicly available NTHi whole-genome sequences were retrieved from the NCBI database and analyzed together with the three newly sequenced genomes. These public datasets were generated using Illumina or PacBio RSII platforms. Although these assemblies varied in contiguity and completeness, their quality metrics (**Table 2**) were considered sufficient and comparable for downstream analyses. Consistent with previous studies, once moderate sequencing depth thresholds are achieved, variation in coverage is unlikely to substantially influence gene presence–absence–based analyses, although fragmented draft genomes may lead to conservative estimates of accessory gene content (Land et al., 2015; Page et al., 2015).

### MLST based molecular typing and population structure of NTHi isolates

**Figure 1:**
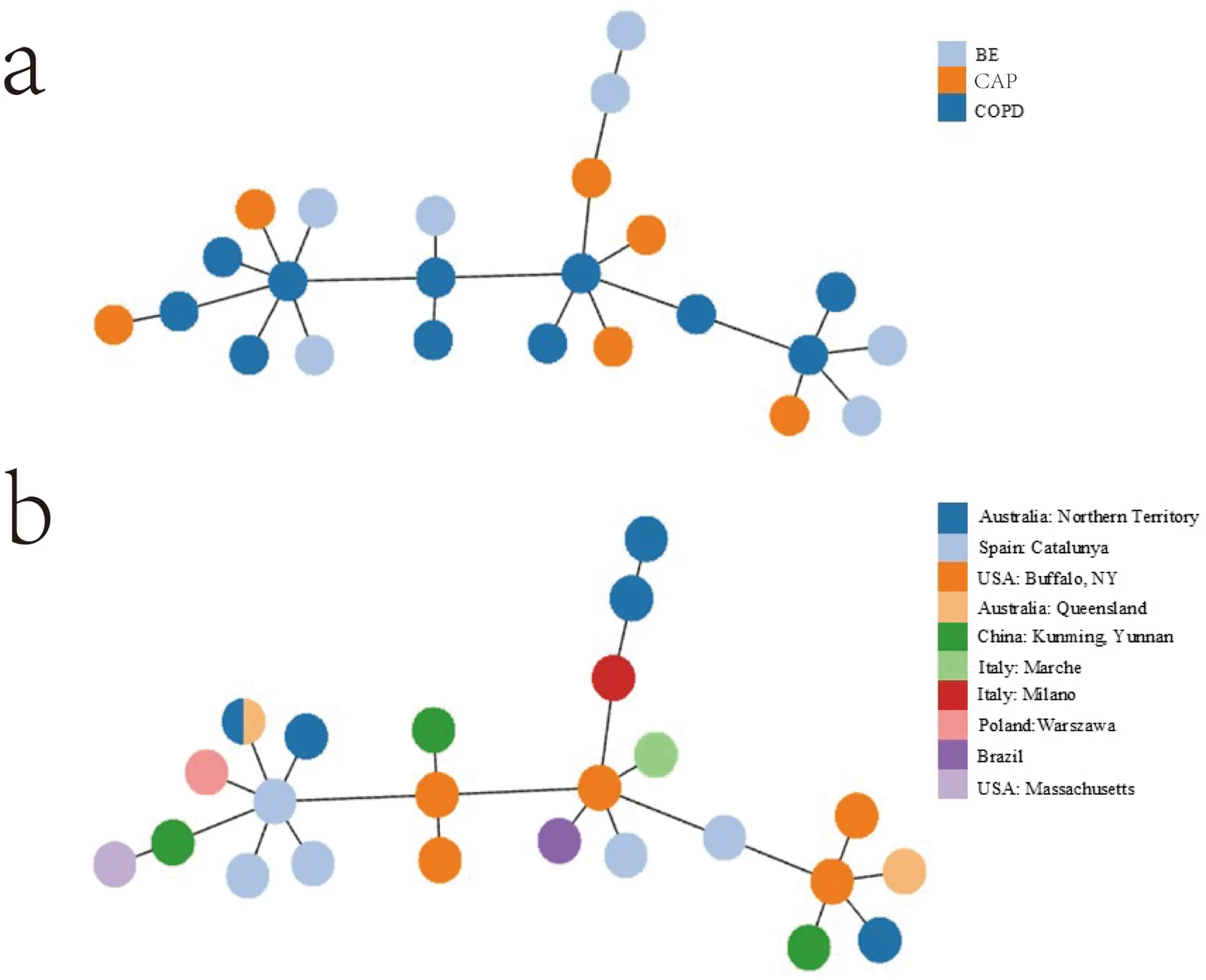
MSTs of 27 NTHi isolates based on MLST profiles. Each node represents a unique ST, and the size of the node is proportional to the number of isolates sharing that ST. Edges indicate allelic relatedness between STs as inferred by the goeBURST algorithm. **(a)** MST colored according to clinical source, including bronchiectasis (BE), CAP, and COPD. **(b)** MST colored according to geographic origin.

A total of 27 NTHi isolates, including three newly sequenced clinical isolates, were included in the whole-genome analysis. To provide an initial assessment of genetic diversity, sequence types (STs) were determined using in silico MLST based on allelic profiles (**Table 3**). MLST analysis showed that the 27 isolates were distributed across 23 distinct STs (**Figure 1a**). Among these, seven STs were identified among bronchiectasis-derived isolates, six among CAP derived isolates, and eleven among COPD derived isolates. Three STs contained more than one isolate, including ST1907 (n = 2), ST1909 (n = 2), and ST1221 (n = 2), whereas the remaining STs were represented by single isolates, indicating substantial genetic diversity within the dataset.

**Table 3:** Differentially distributed accessory genes across clinical groups.

Region_type	Gene	Gene_name	Function
Accessory genes showing differential frequency patterns between bronchiectasis and CAP isolates			
Higher frequency in bronchiectasis	hgpA~~~hgpA_2~~~hgpA_1	hgpA	Virulence factor
Higher frequency in bronchiectasis	zntR	zntR	HTH-type transcriptional regulator ZntR
Higher frequency in CAP	ahpC	ahpC	Virulence factor
Higher frequency in CAP	glyE	glyE	Glycosyl transferase family 8; involved in polysaccharide synthesis
Higher frequency in CAP	*group_545*	*YbaB*	Nucleoid-associated DNA-binding protein
Higher frequency in CAP	*group_473*	*HI_0983*	Hypothetical protein
Accessory genes showing differential frequency patterns between bronchiectasis and COPD isolates			
Higher frequency in bronchiectasis	group_805	icsA	Virulence factor
Higher frequency in bronchiectasis	vapD	vapD	Endoribonuclease VapD; virulence factor
Higher frequency in bronchiectasis	hhaIM	haeIIM	DNA methyltransferase;restriction-modification system
Higher frequency in bronchiectasis	group_720	topA	DNA topoisomerase 1; transcription and replication; biofilm-associated
Higher frequency in bronchiectasis	group_1267	ssb_2	Single-stranded DNA-binding protein
Higher frequency in COPD	group_1188	comB	COMB_HAEIN Competence protein B
Higher frequency in COPD	group_477	molA	Molybdate-binding protein MolA
Higher frequency in COPD	group_931	HI_1568	Mu-like prophage FluMu G protein 2
Higher frequency in COPD	group_640	HI_1324	Lon protease homolog
Higher frequency in COPD	group_333	HI_1667	L, D-transpeptidase
Higher frequency in COPD	group_412	tolA	Tol-Pal system protein TolA; outer membrane integrity; biofilm formation
Higher frequency in COPD	btuC	btuC	Virulence factor
Higher frequency in COPD	group_1102	hifA	Virulence factor
Accessory genes showing differential frequency patterns between CAP and COPD isolates			
Higher frequency in COPD	torY	torY	Cytochrome c-type protein TorY
Higher frequency in COPD	torZ_1~~~torZ~~~torZ_2	torZ	Trimethylamine-N-oxide reductase 2
Higher frequency in COPD	group_972	hpaIIM	Bsp6I methylase
Higher frequency in COPD	tufB~~~tufB_2~~~tufB_1	tufB	Elongation factor Tu 2 in polysaccharide synthesis
Higher frequency in COPD	glmS_1~~~glmS_2	glmS	Glutamate isomerase

Gene clusters containing “~~~” represent merged orthologous gene groups generated by Panaroo during graph-based correction of paralogous and fragmented gene sequences.

CAP, Community-acquired pneumonia; COPD, Chronic Obstructive Pulmonary Disease.

The MST of all isolates was generated using the goeBURST algorithm implemented in PHYLOViZ based on MLST profiles (**Figure 1**) (Francisco et al., 2009, 2012). The resulting MST topology was annotated with metadata describing the geographical (**Figure 1b**) and clinical (**Figure 1a**) origins of the isolates.

Overall, NTHi isolates were broadly dispersed throughout the MST. Isolates from different geographical regions and clinical origins were distributed across multiple branches rather than forming discrete groupings. No clear clustering pattern associated with clinical source was evident. However, it should be noted that the relatively small sample size may have limited the statistical power to detect subtle clustering patterns, particularly given the high genomic diversity of NTHi, and therefore the absence of clear clustering should be interpreted with caution. In addition, the isolates analyzed in this study were derived from multiple countries, diverse anatomical sources (including bronchoalveolar lavage, nasopharyngeal, and blood samples), and a combination of newly sequenced complete genomes and publicly available draft assemblies. These factors may independently influence the observed genetic patterns and are not necessarily linked to clinical disease categories. Therefore, the absence of clustering by clinical phenotype should be interpreted cautiously, and no direct association between MLST-defined population structure and disease type (bronchiectasis, CAP, or COPD) can be inferred from the current dataset.

### Pan-genome architecture and core and accessory genome-based clustering of NTHi isolates

Comparative genomic analysis of the 27 NTHi isolates identified a pan-genome comprising 3,128 genes. The core genome consisted of 1,424 genes present in at least 95% of isolates, accounting for approximately 78% of the 1,821 genes in the reference NTHi strain 86-028NP (Harrison et al., 2005), but only 46% of the total pan-genome.

The remaining genes constituted the accessory genome, including shell and cloud genes. The relatively large proportion of accessory genes indicates substantial genomic diversity among the analyzed isolates. Consistent with previous reports describing a U-shaped pan-genome distribution in NTHi (De Chiara et al., 2014; Donati et al., 2010), these findings support the presence of a heterogeneous accessory gene pool.

**Figure 2:**
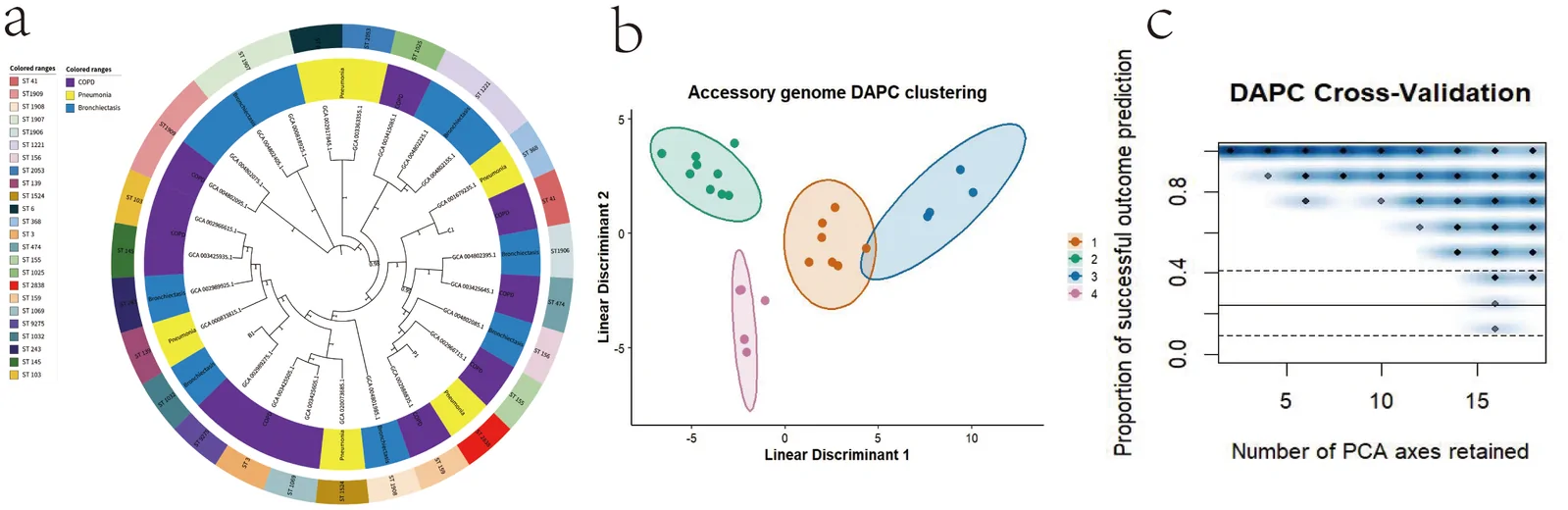
Core genome phylogeny and accessory genome–based DAPC of NTHi isolates. **(a)** Maximum-likelihood phylogenetic tree of 27 NTHi isolates reconstructed from core genome alignment using IQ-TREE v2.2.0. Different colors indicate four genomic clusters inferred from population structure analysis. Numbers at internal nodes represent ultrafast bootstrap support values. Isolates from different clinical groups were distributed across multiple branches of the phylogeny, and no obvious disease-specific clustering pattern was observed. MLST sequence types are overlaid on the phylogeny to enable direct comparison with core genome structure and accessory genome clustering. **(b)** DAPC based on accessory gene presence–absence profiles. **(c)** Cross-validation analysis of accessory genome based DAPC using the xvalDapc function. The plot shows mean assignment success for varying numbers of retained principal components. Two principal components provided the highest mean assignment success, while retention of additional components reduced predictive performance, indicating an increased risk of overfitting at higher dimensionality. These results support the use of a low-dimensional representation for descriptive purposes.

To explore overall genetic relationships, a maximum-likelihood phylogenetic tree was reconstructed from the aligned core genome using IQ-TREE (**Figure 2a**). The resulting phylogeny showed that isolates from different clinical origins were distributed across multiple branches rather than forming distinct groupings, and no clear clustering according to clinical source was observed. STs were additionally annotated on the phylogeny to facilitate comparison with MLST typing, and no obvious concordance between ST distribution and phylogenetic structure was observed.

To further examine variation in accessory genome content, DAPC was performed based on accessory gene presence–absence profiles (**Figure 2b**). BIC analysis suggested a four-cluster solution for exploratory comparison. Under these settings, accessory genome–based clustering showed partial separation among isolates, with limited overlap observed between some inferred clusters, particularly between Clusters I and III. Isolates from different clinical origins were distributed across multiple clusters, and no clear clustering pattern associated with clinical source was observed.

Importantly, the isolates analyzed in this study were derived from multiple geographic regions, diverse anatomical sample types (including bronchoalveolar lavage, nasopharyngeal, and blood), and a combination of newly sequenced complete genomes and publicly available draft assemblies. These factors may independently influence accessory gene presence–absence patterns. Therefore, the observed clustering patterns cannot be attributed specifically to disease categories (bronchiectasis, CAP, or COPD) and should be interpreted as exploratory and descriptive.

Cross-validation analysis using xvalDapc indicated that assignment success increased with the number of retained principal components and reached a plateau at approximately 10 PCs (**Figure 2c**). Retaining additional components provided minimal improvement in discrimination while increasing variability among replicates, suggesting a higher risk of overfitting. Therefore, 10 principal components were retained as a balance between discrimination and model stability for downstream analysis.

### Accessory genome-based DAPC of NTHi isolates across different clinical sources

**Figure 3:**
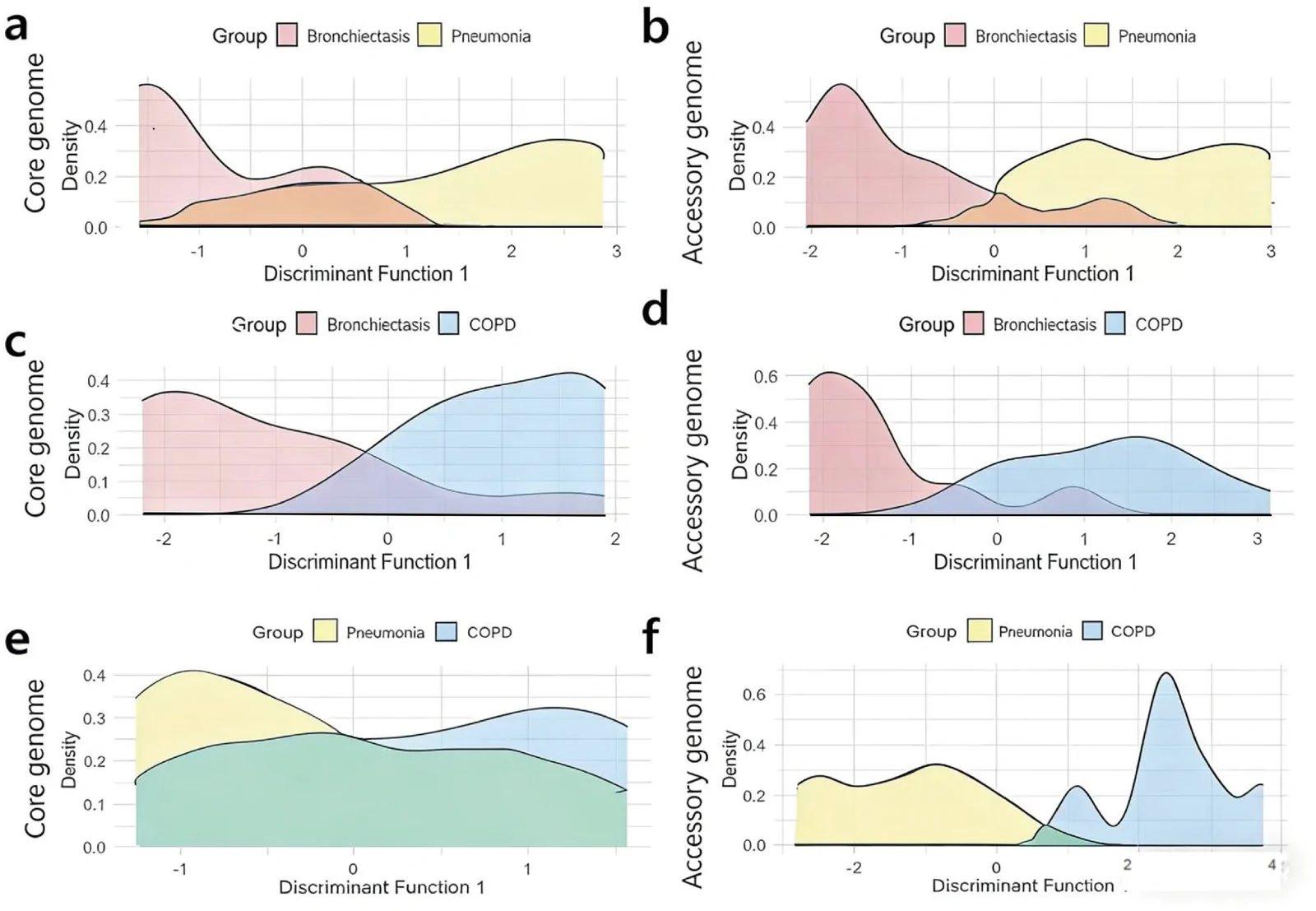
DAPC of 27 NTHi genomes from bronchiectasis, CAP and COPD isolates. **(a)** Discriminant analysis based on cgSNPs reveals substantial overlap between bronchiectasis and CAP along the first discriminant function, indicating poor differentiation between these two phenotypes using core genome polymorphisms. **(b)** In contrast, discriminant analysis based on accessory gene presence/absence profiles demonstrates improved separation, with significantly reduced overlap between bronchiectasis and CAP isolates, suggesting that accessory gene composition has stronger discriminatory power for phenotypic distinction. **(c)** Similarly, cgSNP-based discriminant analysis shows extensive overlap between bronchiectasis and COPD isolates. **(d)** Again, accessory gene-based analysis exhibits better separation with markedly reduced overlapping regions. **(e)** Discriminant analysis based on cgSNPs also shows extensive overlap between CAP and COPD isolates. **(f)** Analysis based on accessory gene presence/absence profiles again demonstrates markedly reduced overlapping regions. Accessory gene composition, rather than core genome polymorphism distribution, serves as the key determinant for distinguishing bronchiectasis isolates from other clinical phenotypes (CAP and COPD).

To further explore accessory genome variation across clinical groups, DAPC based on accessory gene presence–absence profiles was performed. Although substantial overlap remained among isolates from different clinical origins, density distribution analysis of discriminant function 1 scores revealed a partial shift in accessory genome composition between bronchiectasis-derived isolates and isolates from other respiratory disease groups (**Figure 3**).

These findings provide exploratory evidence that accessory genome composition may vary across clinical contexts, although no discrete disease-specific clustering pattern was identified.

### Exploratory pan-genome-wide association analysis of accessory genes

To further explore potential accessory genome variation among respiratory disease phenotypes, a pan-genome-wide association analysis was performed using Scoary (Brynildsrud et al., 2016). Accessory gene presence–absence patterns across a total of 1,714 accessory genes were compared among bronchiectasis, CAP, and COPD isolates.

After Benjamini–Hochberg FDR correction, no accessory genes remained significantly associated for any clinical phenotype. Given the limited sample size and the extensive heterogeneity of the NTHi accessory genome, additional exploratory analyses were performed to identify accessory genes showing differential distribution patterns for descriptive functional interpretation.

Pairwise phenotype comparisons suggested potential differences in gene frequency patterns (Dataset 3). In comparisons between bronchiectasis and CAP, two genes were observed more frequently in bronchiectasis isolates, whereas four were more frequently observed in CAP isolates. In the comparison between bronchiectasis and COPD, 10 and 43 genes showed higher frequencies in bronchiectasis and COPD isolates, respectively. In the CAP versus COPD comparison, nine genes were similarly observed to be more frequent in COPD associated isolates.

### Functional classification of exploratory accessory genes

**Figure 4:**
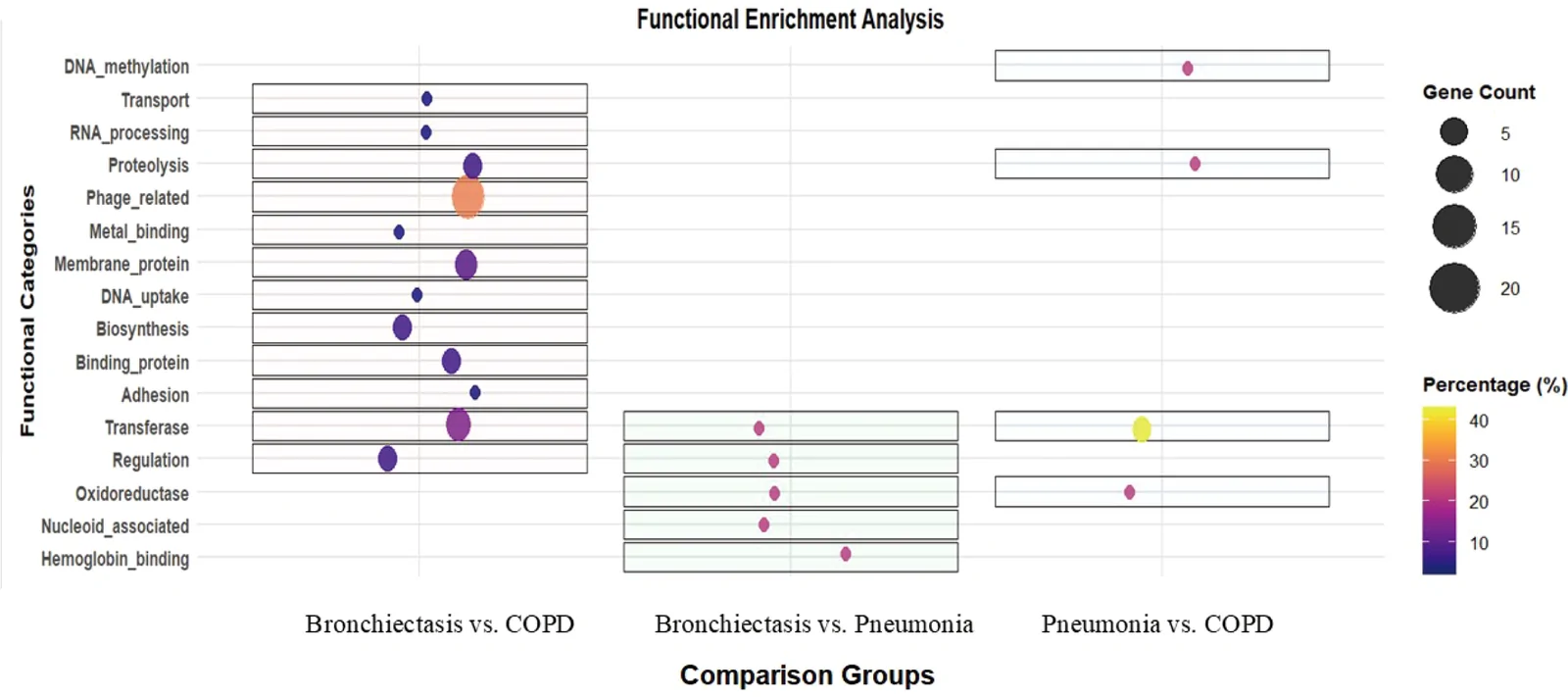
Functional classification of exploratory accessory genes identified by pan-genome-wide association analysis. The bubble plot illustrates the distribution of functional gene categories in isolates from three pairwise clinical comparisons (bronchiectasis vs. COPD, bronchiectasis vs. CAP, and CAP vs. COPD). Bubble size represents the number of genes assigned to each functional category, while color intensity indicates the relative percentage of each function within the corresponding comparison group.

Functional annotation was performed for the 68 exploratory candidate accessory genes identified through pan-genome-wide association analysis. Among these, 53 genes could be assigned to UniProt entries with a minimum sequence identity of 70% (Dataset 4). These genes were distributed across multiple functional categories (**Figure 4**).

In the comparison between bronchiectasis and CAP, six genes showed differences in observed frequency across groups. Two genes (hgpA (Jin et al., 1999) and zntR (Ma et al., 2025)) were more frequently detected in bronchiectasis-associated isolates, whereas four genes (group_545, group_473, ahpC (Cha et al., 2004), and glyE) were more frequently detected in CAP-associated isolates. In the comparison between bronchiectasis associated and COPD associated isolates, 53 genes showed variation in distribution across groups. Genes more frequently detected in bronchiectasis-associated isolates were annotated with functions related to phage-associated processes, DNA recombination, and DNA repair, whereas those more frequently detected in COPD-associated isolates were annotated with functions related to metabolic processes, nutrient uptake, and membrane transport. In the comparison between CAP and COPD isolates, nine genes were more frequently detected in COPD-associated isolates. Functional annotation indicated that these genes were annotated to categories including cellular respiration, epigenetic regulation, protein translation, and cell wall precursor synthesis (**Table 3**).

Additional classification using COG assigned 44 genes to known orthologous categories (Tatusov et al., 2001) but did not substantially extend functional annotation beyond the UniProt-based results (Dataset 2).

## Discussion

In this study, comparative genomic analysis of 27 NTHi isolates revealed substantial genetic diversity, with isolates from bronchiectasis, CAP, and COPD distributed across multiple lineages rather than forming disease-specific clusters. MLST profiles showed broadly consistent grouping with the core genome phylogeny at a coarse phylogenetic level (**Figure 2a**), reflecting the lower resolution of MLST compared with whole-genome approaches. Overall, no clear association between phylogenetic structure and clinical source was observed. The absence of disease-specific clustering is consistent with previous studies reporting high genetic diversity and frequent homologous recombination in NTHi populations, which complicates the identification of stable genotype–phenotype associations (De Chiara et al., 2014; Pinto et al., 2019; Murphy et al., 2023). These evolutionary processes result in extensive genetic mixing, leading to interspersed distribution of isolates from different clinical backgrounds within phylogenetic trees.

Analysis of accessory genome composition using DAPC revealed modest structure among isolates; however, this separation was incomplete and not associated with clinical origin. This discrepancy between core genome phylogeny and accessory genome clustering reflects their distinct evolutionary dynamics, with core genome variation primarily driven by vertical inheritance and accessory genome content shaped by horizontal gene transfer and gene gain–loss processes. Importantly, these patterns should be interpreted cautiously. The dataset includes isolates derived from multiple geographic regions, different anatomical sources, and a combination of newly sequenced genomes and publicly available draft assemblies. Such heterogeneity may substantially influence accessory genome composition and gene presence–absence patterns, independent of biological differences between disease groups.

Pan-genome-wide association analysis did not identify any genes remaining statistically significant after multiple testing correction. Although a small number of genes showed differences in frequency between clinical groups, these findings are exploratory and may reflect sampling variation, genome quality differences, or other confounding factors rather than true disease-associated signals. Greater apparent differentiation was observed in the comparison between bronchiectasis- and COPD-associated isolates; however, these differences likely reflect distributional variation in accessory genes rather than functional adaptation to specific disease states. Functional categorization of differential genes did not reveal enrichment in any dominant biological pathway, but instead showed broad distribution across metabolism, transport, recombination, and phage-related functions. This functional heterogeneity is consistent with the known genomic plasticity of NTHi.

This study has several limitations. First, the relatively small sample size limits statistical power and reduces the ability to detect subtle genotype–phenotype associations. Second, the heterogeneous nature of the dataset—including differences in geographic origin, clinical sampling sources, sequencing platforms, and genome assembly quality—introduces potential biases in accessory genome inference. In particular, the inclusion of draft genomes may lead to underestimation of accessory gene content due to fragmentation. Third, the inherent heterogeneity of clinical categories such as bronchiectasis and COPD may further obscure biological signals. Future studies using larger, more systematically collected datasets with standardized sequencing and assembly pipelines will be essential to reduce technical variability and improve comparability, including consistent genome inclusion criteria, balanced sampling across clinical and geographic sources, and the use of uniformly processed high-quality assemblies. In addition, more refined clinical stratification and integration of multi-omics approaches may help to better resolve the relationship between genomic variation and disease-associated phenotypes in NTHi.

## Conclusion

In conclusion, comparative genomic analysis of NTHi isolates from bronchiectasis, CAP, and COPD indicated that clinical heterogeneity was not reflected in core genome phylogeny. Differences in accessory gene content were observed across isolates from different clinical sources; however, these patterns were heterogeneous and not supported by statistically robust associations after multiple testing correction. Accordingly, no definitive link can be established between accessory genome variation and clinical phenotype in this dataset. These findings should therefore be interpreted as descriptive of genomic diversity rather than evidence of disease-specific adaptation.

## Data Availability

The datasets presented in this study can be found in online repositories. The names of the repository/repositories and accession number(s) can be found in the article/**Supplementary Material**.
